# (1-Acetyl-2,6-diphenyl­piperidin-4-yl­idene)(phen­yl)acetonitrile

**DOI:** 10.1107/S160053680800860X

**Published:** 2008-04-04

**Authors:** A. Manimekalai, A. Balamurugan, S. Balamurugan, A. Thiruvalluvar, R. J. Butcher

**Affiliations:** a Department of Chemistry, Annamalai University, Annamalai Nagar 608 002, Tamilnadu, India; b PG Department of Physics, Rajah Serfoji Govt. College (Autonomous), Thanjavur 613 005, Tamilnadu, India; c Department of Chemistry, Howard University, 525 College Street NW, Washington, DC 20059, USA

## Abstract

In the title mol­ecule, C_27_H_24_N_2_O, the piperidine ring adopts a boat conformation. The acetyl group at position 1 has a bis­ectional orientation. The two phenyl rings attached to the piperidine ring at positions 2 and 6 have bis­ectional and axial orientations, respectively, and make a dihedral angle of 75.27 (10)°. The phenyl­acetonitrile group at position 4 has an equatorial orientation. Mol­ecules are linked by C—H⋯N, C—H⋯O inter­molecular and C—H⋯π inter­actions. A C—H⋯O intra­molecular inter­action is also found in the mol­ecule.

## Related literature

Thiruvalluvar *et al.* (2007 ▶) have reported the crystal structure of (2,6-diphenyl­piperidin-4-yl­idene)(phen­yl)acetonitrile, in which the piperidine ring adopts a chair conformation.
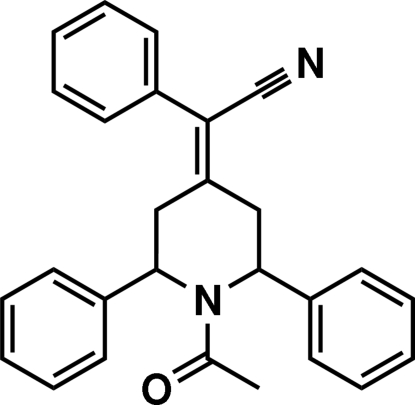


## Experimental

### 

#### Crystal data

C_27_H_24_N_2_O
*M* *_r_* = 392.48
Triclinic, 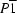

*a* = 9.4034 (17) Å
*b* = 10.646 (6) Å
*c* = 10.8860 (18) Å
α = 90.45 (2)°
β = 99.957 (14)°
γ = 101.98 (3)°
*V* = 1048.9 (7) Å^3^
*Z* = 2
Mo *K*α radiation
μ = 0.08 mm^−1^
*T* = 200 (2) K
0.43 × 0.37 × 0.23 mm

#### Data collection

Oxford Diffraction Gemini diffractometer
Absorption correction: none
14102 measured reflections
6736 independent reflections
2238 reflections with *I* > 2σ(*I*)
*R*_int_ = 0.062

#### Refinement

*R*[*F* ^2^ > 2σ(*F* ^2^)] = 0.054
*wR*(*F* ^2^) = 0.150
*S* = 0.86
6736 reflections
271 parameters
H-atom parameters constrained
Δρ_max_ = 0.17 e Å^−3^
Δρ_min_ = −0.18 e Å^−3^

### 

Data collection: *CrysAlis CCD* (Oxford Diffraction, 2007 ▶); cell refinement: *CrysAlis CCD*; data reduction: *CrysAlis RED* (Oxford Diffraction, 2007 ▶); program(s) used to solve structure: *SHELXS97* (Sheldrick, 2008 ▶); program(s) used to refine structure: *SHELXL97* (Sheldrick, 2008 ▶); molecular graphics: *ORTEP-3* (Farrugia, 1997 ▶); software used to prepare material for publication: *PLATON* (Spek, 2003 ▶).

## Supplementary Material

Crystal structure: contains datablocks global, I. DOI: 10.1107/S160053680800860X/wn2248sup1.cif

Structure factors: contains datablocks I. DOI: 10.1107/S160053680800860X/wn2248Isup2.hkl

Additional supplementary materials:  crystallographic information; 3D view; checkCIF report

## Figures and Tables

**Table 1:** Hydrogen-bond geometry (Å, °)

*D*—H⋯*A*	*D*—H	H⋯*A*	*D*⋯*A*	*D*—H⋯*A*
C6—H6⋯O11	1.00	2.21	2.723 (3)	110
C12—H12*A*⋯N16^i^	0.98	2.50	3.423 (3)	158
C42—H42⋯O11^ii^	0.95	2.58	3.398 (3)	145
C22—H22⋯*Cg*1	0.95	2.79	3.734 (3)	174
C26—H26⋯*Cg*2^iii^	0.95	2.89	3.784 (3)	157

Symmetry codes: (i) 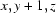; (ii) 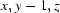; (iii) 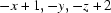. *Cg*1 and *Cg*2 are the centroids of the C61–C66 and C41–C46 phenyl rings, respectively.

**Fig. 1.**
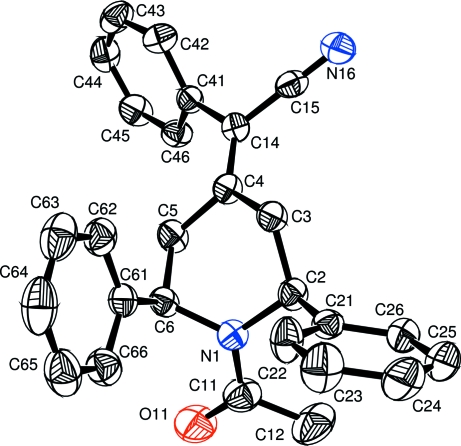
The molecular structure of the title compound, showing the atom-numbering scheme and displacement ellipsoids drawn at the 50% probability level. H atoms have been omitted for clarity.

**Fig. 2.**
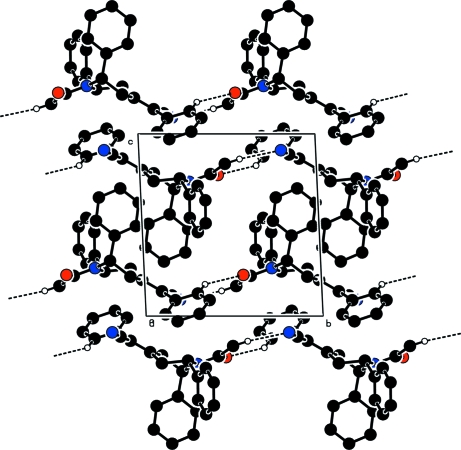
The molecular packing of the title compound, viewed down the a axis. Dashed lines indicate hydrogen bonds. H atoms not involved in hydrogen bonding have been omitted.

## supplementary crystallographic information

### Comment

In the title compound, (Fig. 1), the piperidine ring adopts a boat conformation. The acetyl group at position 1 has a bisectional orientation. The two phenyl rings attached to the piperidine ring at positions 2 and 6 have bisectional and axial orientations, respectively, and make a dihedral angle of 75.27 (10)°. The phenyl-acetonitrile group at position 4 has an equatorial orientation. Molecules are linked by intermolecular C12—H12A···N16, C42—H42···O11 and intramolecular C6—H6···O11 hydrogen bonds. There are C22—H22···π (*x*, *y*, *z*) interactions involving the phenyl ring at 6 and C26—H26···π (1 - *x*, -*y*, 2 - *z*) interactions involving the phenyl ring at C14.

### Experimental

A mixture of (2,6-diphenylpiperidin-4-ylidene)(phenyl)acetonitrile (3.5 g, 0.01 mol), acetic anhydride (2.8 ml, 0.03 mol) and trimethylamine (4.2 ml, 0.03 mol) in benzene (50 ml) was refluxed for 8-10 h. The reaction mixture was cooled to room temperature and poured into ice-cold water. The solid mass was separated by filtration, dried and recrystallized from ethanol. The yield of the isolated product was 2.16 g (55%).

### Refinement

The C-bound H atoms were positioned geometrically and allowed to ride on their parent atoms with C—H = 0.95–1.00 Å and *U*_iso_(H) = 1.2–1.5*U*_eq_(parent atom).

### Crystal data

**Table:**

C_27_H_24_N_2_O	*Z* = 2
*M**_r_* = 392.48	*F*_000_ = 416
Triclinic, *P*1	*D*_x_ = 1.243 Mg m^−^^3^
Hall symbol: -P 1	Melting point: 411 K
*a* = 9.4034 (17) Å	Mo *K*α radiation λ = 0.71073 Å
*b* = 10.646 (6) Å	Cell parameters from 7731 reflections
*c* = 10.8860 (18) Å	θ = 4.5–32.5º
α = 90.45 (2)º	µ = 0.08 mm^−^^1^
β = 99.957 (14)º	*T* = 200 (2) K
γ = 101.98 (3)º	Prism, colourless
*V* = 1048.9 (7) Å^3^	0.43 × 0.37 × 0.23 mm

### Data collection

**Table:**

Oxford Diffraction Gemini diffractometer	6736 independent reflections
Radiation source: fine-focus sealed tube	2238 reflections with *I* > 2σ(*I*)
Monochromator: graphite	*R*_int_ = 0.062
Detector resolution: 10.5081 pixels mm^-1^	θ_max_ = 32.4º
*T* = 200(2) K	θ_min_ = 4.5º
φ and ω scans	*h* = −14→13
Absorption correction: none	*k* = −15→15
14102 measured reflections	*l* = −16→14

### Refinement

**Table:**

Refinement on *F*^2^	Secondary atom site location: difference Fourier map
Least-squares matrix: full	Hydrogen site location: inferred from neighbouring sites
*R*[*F*^2^ > 2σ(*F*^2^)] = 0.055	H-atom parameters constrained
*wR*(*F*^2^) = 0.151	*w* = 1/[σ^2^(*F*_o_^2^) + (0.0581*P*)^2^] where *P* = (*F*_o_^2^ + 2*F*_c_^2^)/3
*S* = 0.86	(Δ/σ)_max_ < 0.001
6736 reflections	Δρ_max_ = 0.17 e Å^−^^3^
271 parameters	Δρ_min_ = −0.18 e Å^−^^3^
Primary atom site location: structure-invariant direct methods	Extinction correction: none

### Special details

**Table:**

Geometry. Bond distances, angles *etc*. have been calculated using the rounded fractional coordinates. All su's are estimated from the variances of the (full) variance-covariance matrix. The cell e.s.d.'s are taken into account in the estimation of distances, angles and torsion angles
Refinement. Refinement of *F*^2^ against ALL reflections. The weighted *R*-factor *wR* and goodness of fit *S* are based on *F*^2^, conventional *R*-factors *R* are based on *F*, with *F* set to zero for negative *F*^2^. The threshold expression of *F*^2^ > σ(*F*^2^) is used only for calculating *R*-factors(gt) *etc*. and is not relevant to the choice of reflections for refinement. *R*-factors based on *F*^2^ are statistically about twice as large as those based on *F*, and *R*- factors based on ALL data will be even larger.

### Fractional atomic coordinates and isotropic or equivalent isotropic displacement parameters (Å^2^)

**Table:**

	*x*	*y*	*z*	*U*_iso_*/*U*_eq_	
O11	0.28221 (18)	0.44042 (15)	0.77415 (18)	0.0685 (7)	
N1	0.39382 (16)	0.27784 (16)	0.73697 (15)	0.0349 (6)	
N16	0.59278 (19)	−0.19597 (17)	0.91049 (19)	0.0506 (7)	
C2	0.52978 (19)	0.22457 (18)	0.75539 (19)	0.0359 (7)	
C3	0.49051 (19)	0.07647 (18)	0.7407 (2)	0.0360 (7)	
C4	0.36419 (19)	0.01309 (19)	0.80179 (18)	0.0328 (6)	
C5	0.2347 (2)	0.07931 (19)	0.7838 (2)	0.0398 (7)	
C6	0.24863 (19)	0.19042 (18)	0.69692 (19)	0.0342 (7)	
C11	0.3964 (3)	0.3991 (2)	0.7794 (2)	0.0516 (9)	
C12	0.5447 (3)	0.4833 (2)	0.8355 (3)	0.0754 (10)	
C14	0.36214 (19)	−0.09522 (18)	0.86415 (17)	0.0298 (6)	
C15	0.4908 (2)	−0.15071 (18)	0.8868 (2)	0.0374 (7)	
C21	0.6360 (2)	0.27610 (18)	0.6675 (2)	0.0345 (7)	
C22	0.5903 (2)	0.2716 (2)	0.5393 (2)	0.0479 (8)	
C23	0.6893 (2)	0.3135 (2)	0.4608 (2)	0.0584 (9)	
C24	0.8362 (2)	0.3599 (2)	0.5095 (3)	0.0554 (10)	
C25	0.8845 (2)	0.3641 (2)	0.6354 (3)	0.0526 (9)	
C26	0.7842 (2)	0.32381 (19)	0.7149 (2)	0.0440 (7)	
C41	0.23021 (19)	−0.16901 (18)	0.91288 (19)	0.0325 (7)	
C42	0.1746 (2)	−0.29754 (19)	0.87607 (19)	0.0394 (7)	
C43	0.0464 (2)	−0.3626 (2)	0.9124 (2)	0.0479 (8)	
C44	−0.0256 (2)	−0.3021 (2)	0.9879 (2)	0.0505 (8)	
C45	0.0332 (2)	−0.1759 (2)	1.0289 (2)	0.0485 (8)	
C46	0.1591 (2)	−0.1095 (2)	0.9908 (2)	0.0385 (7)	
C61	0.21670 (19)	0.1583 (2)	0.5582 (2)	0.0360 (7)	
C62	0.1943 (2)	0.0355 (2)	0.5061 (2)	0.0449 (8)	
C63	0.1684 (2)	0.0136 (3)	0.3767 (3)	0.0568 (9)	
C64	0.1629 (2)	0.1141 (3)	0.2993 (2)	0.0615 (9)	
C65	0.1818 (2)	0.2372 (3)	0.3491 (2)	0.0593 (10)	
C66	0.2075 (2)	0.2592 (2)	0.4772 (2)	0.0466 (8)	
H2	0.58301	0.24860	0.84282	0.0430*	
H3A	0.46581	0.05133	0.65055	0.0431*	
H3B	0.57893	0.04320	0.77637	0.0431*	
H5A	0.22351	0.11179	0.86628	0.0477*	
H5B	0.14360	0.01501	0.75020	0.0477*	
H6	0.17358	0.23999	0.71251	0.0411*	
H12A	0.53045	0.56738	0.86194	0.1132*	
H12B	0.60930	0.49466	0.77301	0.1132*	
H12C	0.59019	0.44235	0.90790	0.1132*	
H22	0.48916	0.23918	0.50488	0.0576*	
H23	0.65600	0.31034	0.37307	0.0701*	
H24	0.90419	0.38900	0.45537	0.0665*	
H25	0.98632	0.39436	0.66885	0.0632*	
H26	0.81777	0.32916	0.80261	0.0528*	
H42	0.22454	−0.34069	0.82602	0.0472*	
H43	0.00736	−0.44986	0.88522	0.0575*	
H44	−0.11457	−0.34695	1.01136	0.0606*	
H45	−0.01332	−0.13461	1.08343	0.0582*	
H46	0.19769	−0.02222	1.01816	0.0462*	
H62	0.19645	−0.03492	0.55895	0.0539*	
H63	0.15464	−0.07120	0.34214	0.0681*	
H64	0.14607	0.09897	0.21134	0.0736*	
H65	0.17710	0.30679	0.29547	0.0710*	
H66	0.21918	0.34412	0.51091	0.0559*	

### Atomic displacement parameters (Å^2^)

**Table:**

	*U*^11^	*U*^22^	*U*^33^	*U*^12^	*U*^13^	*U*^23^
O11	0.0661 (10)	0.0515 (10)	0.0951 (14)	0.0227 (9)	0.0221 (10)	−0.0108 (10)
N1	0.0359 (9)	0.0358 (10)	0.0346 (10)	0.0086 (7)	0.0099 (7)	−0.0008 (8)
N16	0.0456 (10)	0.0407 (11)	0.0677 (14)	0.0131 (9)	0.0114 (10)	0.0047 (10)
C2	0.0304 (10)	0.0402 (12)	0.0383 (13)	0.0066 (9)	0.0108 (9)	0.0049 (10)
C3	0.0303 (10)	0.0367 (12)	0.0438 (13)	0.0077 (9)	0.0137 (9)	0.0091 (10)
C4	0.0310 (10)	0.0365 (11)	0.0328 (12)	0.0059 (9)	0.0125 (8)	0.0041 (9)
C5	0.0329 (10)	0.0479 (13)	0.0423 (13)	0.0103 (10)	0.0146 (9)	0.0109 (11)
C6	0.0292 (10)	0.0376 (11)	0.0397 (13)	0.0105 (9)	0.0121 (9)	0.0085 (10)
C11	0.0546 (14)	0.0438 (14)	0.0600 (17)	0.0117 (12)	0.0191 (12)	−0.0015 (12)
C12	0.0726 (17)	0.0482 (16)	0.101 (2)	0.0018 (13)	0.0184 (16)	−0.0301 (16)
C14	0.0325 (10)	0.0347 (11)	0.0228 (10)	0.0075 (9)	0.0061 (8)	0.0022 (9)
C15	0.0399 (12)	0.0275 (11)	0.0457 (14)	0.0031 (10)	0.0153 (10)	0.0007 (10)
C21	0.0335 (10)	0.0305 (11)	0.0414 (14)	0.0070 (9)	0.0116 (9)	0.0044 (9)
C22	0.0330 (11)	0.0689 (16)	0.0402 (14)	0.0046 (11)	0.0092 (10)	0.0063 (12)
C23	0.0523 (14)	0.0773 (18)	0.0452 (15)	0.0045 (13)	0.0183 (12)	0.0131 (14)
C24	0.0482 (14)	0.0574 (16)	0.0634 (19)	0.0019 (11)	0.0281 (12)	0.0153 (13)
C25	0.0355 (11)	0.0498 (14)	0.072 (2)	0.0028 (10)	0.0153 (12)	0.0101 (13)
C26	0.0364 (11)	0.0425 (13)	0.0510 (14)	0.0051 (10)	0.0054 (10)	0.0079 (11)
C41	0.0291 (10)	0.0335 (12)	0.0355 (12)	0.0065 (9)	0.0072 (9)	0.0065 (10)
C42	0.0503 (12)	0.0353 (12)	0.0328 (12)	0.0053 (10)	0.0126 (10)	0.0031 (10)
C43	0.0536 (13)	0.0393 (13)	0.0434 (15)	−0.0058 (11)	0.0069 (11)	0.0087 (11)
C44	0.0375 (12)	0.0583 (16)	0.0519 (15)	−0.0005 (11)	0.0096 (11)	0.0246 (13)
C45	0.0451 (12)	0.0513 (15)	0.0562 (15)	0.0147 (11)	0.0229 (11)	0.0153 (12)
C46	0.0409 (11)	0.0354 (12)	0.0417 (13)	0.0081 (10)	0.0140 (10)	0.0088 (10)
C61	0.0245 (10)	0.0401 (12)	0.0454 (14)	0.0078 (9)	0.0102 (9)	0.0036 (11)
C62	0.0332 (11)	0.0490 (14)	0.0524 (16)	0.0067 (10)	0.0097 (10)	−0.0003 (12)
C63	0.0429 (13)	0.0673 (17)	0.0575 (18)	0.0074 (12)	0.0080 (12)	−0.0222 (15)
C64	0.0398 (13)	0.101 (2)	0.0390 (15)	0.0043 (14)	0.0071 (11)	−0.0031 (16)
C65	0.0472 (14)	0.081 (2)	0.0468 (17)	0.0102 (13)	0.0042 (12)	0.0192 (15)
C66	0.0418 (12)	0.0504 (14)	0.0487 (15)	0.0116 (10)	0.0087 (10)	0.0068 (12)

### Geometric parameters (Å, °)

**Table:**

O11—C11	1.235 (3)	C62—C63	1.397 (4)
N1—C2	1.487 (3)	C63—C64	1.371 (4)
N1—C6	1.477 (3)	C64—C65	1.379 (4)
N1—C11	1.362 (3)	C65—C66	1.383 (3)
N16—C15	1.152 (3)	C2—H2	1.0000
C2—C3	1.543 (3)	C3—H3A	0.9900
C2—C21	1.525 (3)	C3—H3B	0.9900
C3—C4	1.502 (3)	C5—H5A	0.9900
C4—C5	1.515 (3)	C5—H5B	0.9900
C4—C14	1.341 (3)	C6—H6	1.0000
C5—C6	1.519 (3)	C12—H12A	0.9800
C6—C61	1.510 (3)	C12—H12B	0.9800
C11—C12	1.516 (4)	C12—H12C	0.9800
C14—C15	1.439 (3)	C22—H22	0.9500
C14—C41	1.506 (3)	C23—H23	0.9500
C21—C22	1.385 (3)	C24—H24	0.9500
C21—C26	1.386 (3)	C25—H25	0.9500
C22—C23	1.380 (3)	C26—H26	0.9500
C23—C24	1.376 (3)	C42—H42	0.9500
C24—C25	1.365 (5)	C43—H43	0.9500
C25—C26	1.394 (3)	C44—H44	0.9500
C41—C42	1.389 (3)	C45—H45	0.9500
C41—C46	1.390 (3)	C46—H46	0.9500
C42—C43	1.383 (3)	C62—H62	0.9500
C43—C44	1.383 (3)	C63—H63	0.9500
C44—C45	1.381 (3)	C64—H64	0.9500
C45—C46	1.379 (3)	C65—H65	0.9500
C61—C62	1.381 (3)	C66—H66	0.9500
C61—C66	1.402 (3)		
			
O11···C42^i^	3.398 (3)	C63···H5B^vi^	2.9700
O11···H6	2.2100	C64···H5B^vi^	2.8800
O11···H25^ii^	2.7600	C65···H22	3.0800
O11···H42^i^	2.5800	C66···H22	2.6700
N16···C12^iii^	3.423 (3)	H2···C12	2.6000
N1···H22	2.8800	H2···H12C	2.1600
N1···H66	2.8900	H2···H26	2.3100
N16···H12A^iii^	2.5000	H3A···C6	2.8700
N16···H5A^iv^	2.7500	H3A···C22	2.7800
N16···H46^iv^	2.7200	H3A···C61	2.8500
C3···C62	3.390 (3)	H3A···C62	2.7300
C3···C61	3.244 (3)	H3A···H22	2.5600
C4···C62	3.371 (3)	H3A···H62	2.5300
C5···C46	3.107 (3)	H3B···C15	2.4600
C12···C26	3.501 (4)	H5A···C41	3.0500
C12···C21	3.196 (4)	H5A···C46	2.7400
C12···N16^i^	3.423 (3)	H5A···H46	2.2000
C21···C12	3.196 (4)	H5A···N16^iv^	2.7500
C22···C66	3.520 (3)	H5A···C45^vii^	3.0400
C22···C61	3.512 (3)	H5A···H45^vii^	2.2000
C26···C45^iv^	3.570 (4)	H5B···C41	2.8000
C26···C12	3.501 (4)	H5B···C46	2.9400
C42···O11^iii^	3.398 (3)	H5B···C62	2.7800
C43···C43^v^	3.544 (4)	H5B···H62	2.3100
C45···C26^iv^	3.570 (4)	H5B···C63^vi^	2.9700
C46···C5	3.107 (3)	H5B···C64^vi^	2.8800
C61···C3	3.244 (3)	H6···O11	2.2100
C61···C22	3.512 (3)	H6···H66	2.5300
C62···C3	3.390 (3)	H12A···N16^i^	2.5000
C62···C62^vi^	3.553 (3)	H12B···C2	2.8100
C62···C4	3.371 (3)	H12B···C21	2.6600
C66···C22	3.520 (3)	H12B···C26	2.8300
C2···H12B	2.8100	H12C···C2	2.7400
C2···H12C	2.7400	H12C···H2	2.1600
C4···H62	2.8200	H22···N1	2.8800
C4···H46	3.0300	H22···C61	2.6900
C5···H45^vii^	2.8800	H22···C65	3.0800
C5···H62	2.6600	H22···C66	2.6700
C5···H46	2.8300	H22···H3A	2.5600
C6···H3A	2.8700	H25···O11^ix^	2.7600
C11···H66	3.0900	H26···H2	2.3100
C12···H2	2.6000	H26···C44^iv^	2.7900
C15···H42	2.8500	H26···C45^iv^	2.8500
C15···H3B	2.4600	H42···O11^iii^	2.5800
C21···H12B	2.6600	H42···C15	2.8500
C22···H3A	2.7800	H43···C43^v^	3.0200
C25···H63^viii^	3.0800	H43···C44^v^	3.0100
C26···H63^viii^	2.9500	H45···C5^vii^	2.8800
C26···H12B	2.8300	H45···H5A^vii^	2.2000
C41···H5B	2.8000	H46···C4	3.0300
C41···H5A	3.0500	H46···C5	2.8300
C43···H65^vi^	2.9600	H46···H5A	2.2000
C43···H43^v^	3.0200	H46···N16^iv^	2.7200
C44···H26^iv^	2.7900	H62···C4	2.8200
C44···H43^v^	3.0100	H62···C5	2.6600
C45···H64^vi^	3.0700	H62···H3A	2.5300
C45···H5A^vii^	3.0400	H62···H5B	2.3100
C45···H26^iv^	2.8500	H63···C25^viii^	3.0800
C46···H5A	2.7400	H63···C26^viii^	2.9500
C46···H5B	2.9400	H64···C45^vi^	3.0700
C61···H22	2.6900	H65···C43^vi^	2.9600
C61···H3A	2.8500	H66···N1	2.8900
C62···H5B	2.7800	H66···C11	3.0900
C62···H3A	2.7300	H66···H6	2.5300
			
C2—N1—C6	119.34 (15)	C2—C3—H3B	109.00
C2—N1—C11	121.28 (18)	C4—C3—H3A	109.00
C6—N1—C11	118.09 (18)	C4—C3—H3B	109.00
N1—C2—C3	110.80 (15)	H3A—C3—H3B	108.00
N1—C2—C21	113.88 (16)	C4—C5—H5A	109.00
C3—C2—C21	108.75 (16)	C4—C5—H5B	109.00
C2—C3—C4	114.81 (16)	C6—C5—H5A	109.00
C3—C4—C5	114.37 (17)	C6—C5—H5B	109.00
C3—C4—C14	123.67 (17)	H5A—C5—H5B	108.00
C5—C4—C14	121.93 (18)	N1—C6—H6	106.00
C4—C5—C6	114.44 (17)	C5—C6—H6	106.00
N1—C6—C5	108.81 (16)	C61—C6—H6	106.00
N1—C6—C61	112.14 (16)	C11—C12—H12A	109.00
C5—C6—C61	117.64 (17)	C11—C12—H12B	110.00
O11—C11—N1	121.8 (2)	C11—C12—H12C	109.00
O11—C11—C12	120.1 (2)	H12A—C12—H12B	109.00
N1—C11—C12	118.1 (2)	H12A—C12—H12C	109.00
C4—C14—C15	120.42 (18)	H12B—C12—H12C	109.00
C4—C14—C41	124.59 (17)	C21—C22—H22	119.00
C15—C14—C41	114.99 (16)	C23—C22—H22	120.00
N16—C15—C14	176.9 (2)	C22—C23—H23	120.00
C2—C21—C22	121.85 (18)	C24—C23—H23	120.00
C2—C21—C26	119.99 (19)	C23—C24—H24	120.00
C22—C21—C26	118.10 (19)	C25—C24—H24	120.00
C21—C22—C23	121.06 (19)	C24—C25—H25	120.00
C22—C23—C24	120.0 (2)	C26—C25—H25	120.00
C23—C24—C25	120.1 (2)	C21—C26—H26	120.00
C24—C25—C26	119.9 (2)	C25—C26—H26	120.00
C21—C26—C25	120.8 (2)	C41—C42—H42	120.00
C14—C41—C42	119.79 (17)	C43—C42—H42	120.00
C14—C41—C46	121.13 (17)	C42—C43—H43	120.00
C42—C41—C46	119.04 (18)	C44—C43—H43	120.00
C41—C42—C43	119.87 (19)	C43—C44—H44	120.00
C42—C43—C44	120.78 (19)	C45—C44—H44	120.00
C43—C44—C45	119.32 (19)	C44—C45—H45	120.00
C44—C45—C46	120.22 (19)	C46—C45—H45	120.00
C41—C46—C45	120.68 (19)	C41—C46—H46	120.00
C6—C61—C62	124.09 (19)	C45—C46—H46	120.00
C6—C61—C66	117.97 (18)	C61—C62—H62	120.00
C62—C61—C66	117.9 (2)	C63—C62—H62	120.00
C61—C62—C63	120.8 (2)	C62—C63—H63	120.00
C62—C63—C64	120.2 (3)	C64—C63—H63	120.00
C63—C64—C65	120.0 (2)	C63—C64—H64	120.00
C64—C65—C66	119.9 (2)	C65—C64—H64	120.00
C61—C66—C65	121.1 (2)	C64—C65—H65	120.00
N1—C2—H2	108.00	C66—C65—H65	120.00
C3—C2—H2	108.00	C61—C66—H66	119.00
C21—C2—H2	108.00	C65—C66—H66	119.00
C2—C3—H3A	109.00		
			
C6—N1—C2—C3	−5.9 (2)	C5—C6—C61—C62	−8.6 (3)
C6—N1—C2—C21	117.10 (19)	C5—C6—C61—C66	170.61 (17)
C11—N1—C2—C3	160.88 (18)	C4—C14—C41—C42	123.0 (2)
C11—N1—C2—C21	−76.2 (2)	C4—C14—C41—C46	−54.6 (3)
C2—N1—C6—C5	51.9 (2)	C15—C14—C41—C42	−55.8 (3)
C2—N1—C6—C61	−80.0 (2)	C15—C14—C41—C46	126.6 (2)
C11—N1—C6—C5	−115.26 (19)	C2—C21—C22—C23	−177.12 (19)
C11—N1—C6—C61	112.9 (2)	C26—C21—C22—C23	−0.1 (3)
C2—N1—C11—O11	−173.7 (2)	C2—C21—C26—C25	176.10 (18)
C2—N1—C11—C12	5.6 (3)	C22—C21—C26—C25	−1.0 (3)
C6—N1—C11—O11	−6.8 (3)	C21—C22—C23—C24	0.4 (3)
C6—N1—C11—C12	172.5 (2)	C22—C23—C24—C25	0.3 (3)
N1—C2—C3—C4	−42.5 (2)	C23—C24—C25—C26	−1.4 (3)
C21—C2—C3—C4	−168.35 (17)	C24—C25—C26—C21	1.7 (3)
N1—C2—C21—C22	−53.0 (2)	C14—C41—C42—C43	−174.69 (19)
N1—C2—C21—C26	130.00 (19)	C46—C41—C42—C43	2.9 (3)
C3—C2—C21—C22	71.1 (2)	C14—C41—C46—C45	176.11 (19)
C3—C2—C21—C26	−105.9 (2)	C42—C41—C46—C45	−1.5 (3)
C2—C3—C4—C5	42.4 (2)	C41—C42—C43—C44	−1.7 (3)
C2—C3—C4—C14	−139.6 (2)	C42—C43—C44—C45	−1.1 (3)
C3—C4—C5—C6	5.6 (2)	C43—C44—C45—C46	2.6 (3)
C14—C4—C5—C6	−172.45 (18)	C44—C45—C46—C41	−1.3 (3)
C3—C4—C14—C15	6.4 (3)	C6—C61—C62—C63	−178.61 (19)
C3—C4—C14—C41	−172.37 (18)	C66—C61—C62—C63	2.2 (3)
C5—C4—C14—C15	−175.75 (18)	C6—C61—C66—C65	178.57 (18)
C5—C4—C14—C41	5.5 (3)	C62—C61—C66—C65	−2.2 (3)
C4—C5—C6—N1	−50.8 (2)	C61—C62—C63—C64	−0.9 (3)
C4—C5—C6—C61	78.1 (2)	C62—C63—C64—C65	−0.5 (3)
N1—C6—C61—C62	118.7 (2)	C63—C64—C65—C66	0.5 (3)
N1—C6—C61—C66	−62.1 (2)	C64—C65—C66—C61	0.8 (3)

Symmetry codes: (i) *x*, *y*+1, *z*; (ii) *x*−1, *y*, *z*; (iii) *x*, *y*−1, *z*; (iv) −*x*+1, −*y*, −*z*+2; (v) −*x*, −*y*−1, −*z*+2; (vi) −*x*, −*y*, −*z*+1; (vii) −*x*, −*y*, −*z*+2; (viii) −*x*+1, −*y*, −*z*+1; (ix) *x*+1, *y*, *z*.

### Hydrogen-bond geometry (Å, °)

**Table:**

*D*—H···*A*	*D*—H	H···*A*	*D*···*A*	*D*—H···*A*
C6—H6···O11	1.00	2.21	2.723 (3)	110
C12—H12A···N16^i^	0.98	2.50	3.423 (3)	158
C42—H42···O11^iii^	0.95	2.58	3.398 (3)	145
C22—H22···Cg1	0.95	2.79	3.734 (3)	174
C26—H26···Cg2^iv^	0.95	2.89	3.784 (3)	157

Symmetry codes: (i) *x*, *y*+1, *z*; (iii) *x*, *y*−1, *z*;  (iv) −*x*+1, −*y*, −*z*+2.
